# Relationship of transcriptional markers to Leydig cell number in the mouse testis

**DOI:** 10.1371/journal.pone.0219524

**Published:** 2019-07-10

**Authors:** Diane Rebourcet, Ana Monteiro, Lyndsey Cruickshanks, Nathan Jeffery, Sarah Smith, Laura Milne, Peter J. O’Shaughnessy, Lee B. Smith

**Affiliations:** 1 Faculty of Science, University of Newcastle, Callaghan, NSW, Australia; 2 Institute of Biodiversity, Animal Health and Comparative Medicine, University of Glasgow, Glasgow, United Kingdom; 3 MRC Centre for Reproductive Health, University of Edinburgh, The Queen’s Medical Research Institute, Edinburgh, EH, United Kingdom; University Hospital of Münster, GERMANY

## Abstract

**Objectives:**

The current study aims to identify markers that would reflect the number of Leydig cells present in the testis, to help determine whether labour-intensive methods such as stereology are necessary. We used our well-characterised Sertoli cell ablation model in which we have empirically established the size of the Leydig cell population, to try to identify transcriptional biomarkers indicative of population size.

**Results:**

Following characterisation of the Leydig cell population after Sertoli cell ablation in neonatal life or adulthood, we identified *Hsd3b1* transcript levels as a potential indicator of Leydig cell number with utility for informing decision-making on whether to engage in time-consuming stereological cell counting analysis.

## Introduction

Androgens are critical for male development, fertility and well-being [1–4]. The principal source of androgens in the male are the testicular Leydig cells (LCs), which develop in two sequential waves or populations. The first population to develop are the fetal Leydig cells (FLCs), which appear soon after testis differentiation and act to ensure masculinisation of the fetus. After birth, a second “adult” Leydig cell (ALC) population starts to develop in the mouse at around 7–10 days [5–7]. These cells generate a pubertal surge of testosterone, which acts to promote and maintain adult fertility and to drive male reproductive behaviour. Our group has recently shown that the Sertoli cells (SCs), key regulators of testis differentiation and adult spermatogenesis, also control both the differentiation and development of the ALC population in neonatal life, and their survival in adulthood [8, 9]. Additionally, SC number was shown to be a biomarker of ALC population size [10]. However, these studies relied on the application of thorough biomolecular and stereological approaches to determine cell population sizes, the latter being somewhat time-consuming. Identification of a biomarker that is predictive of a change in LC population size would speed preliminary analysis, and inform a decision on whether more time-consuming stereological counting is likely to be informative.

To this end, we used our well-characterised SC ablation model in which we have empirically established the size of the LC population to try to identify transcriptional biomarkers indicative of population size. We know that, in this model, SC ablation in adulthood leads to loss of 75% of the LC population. However, the 25% of LCs that remain after SC ablation in the adult are able to maintain basal testosterone levels in the mice although the pulsatile peak levels of hormone are reduced [8], suggesting that the remaining LCs are showing increased levels of activity. Therefore, this provides a unique opportunity to identify LC transcripts that accurately reflect the size of the LC population, as opposed to those that change in response to a functional compensation for a reduction in population size.

To address this, we first determined the characteristics of the LCs following SC ablation, using histological and bio molecular approaches. We then utilised this model to identify gene transcripts that can be utilised as indicators of a change in ALC number for use in future studies.

## Results discussion

### Postnatal retention of fetal-like-LCs is independent of SCs

**Fig 1:**
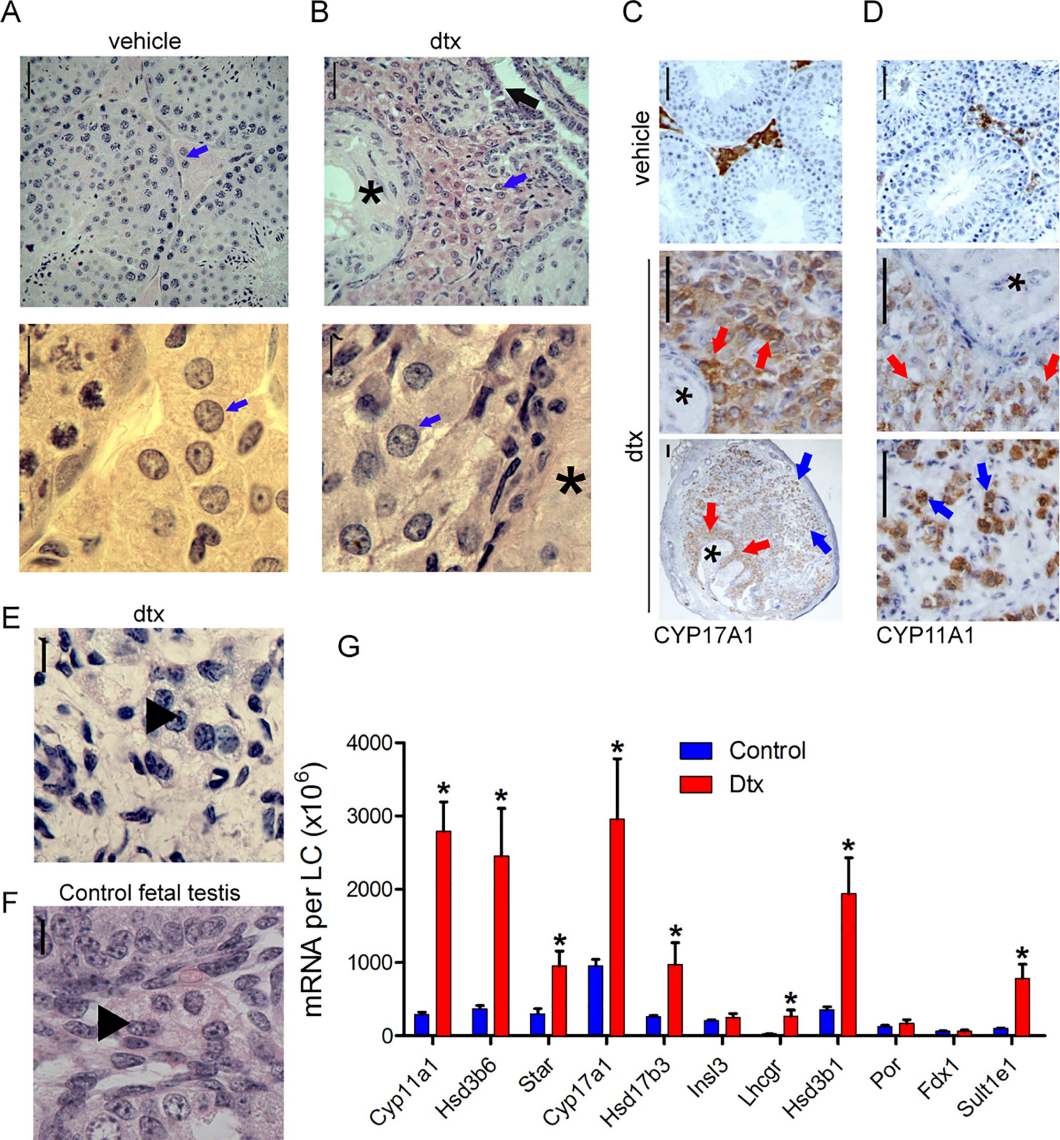
Characterisation of the Leydig cell following Sertoli cell ablation in the neonate. (A) Normal adult testis (postnatal day 80 (pnd80)). Blue arrows indicate Leydig cells. (B) Adult testis (pnd80) following neonatal Sertoli cell ablation. A large cluster of Leydig cells (blue arrows) is present around the tubuli recti (asterix) and rete testis (black arrow). Leydig cells from this group in higher power appear similar to normal adult Leydig cells (A, B blue arrows). Immunolocalisation of CYP17A1 (C) and CYP11A1 (D) following Sertoli cell ablation in the neonate showed Leydig cells clustered around the tubuli recti (asterix). There were also small clusters of cells expressing CYP11A1 and CYP17A1 in the testis parenchyma more distant from the tubuli recti (blue arrows). The morphology of these cells is shown in (E-black arrowhead). For comparison, Leydig cells from a normal neonatal (pnd2) mouse are shown in (F-black arrowhead). These cells have the same morphology as (F). The black bars represent 50μm, apart from higher power images in A) and B) which are 10μm. (G) Transcript levels measured at pnd80. Results have been normalised to the number of Leydig cells present in these mice based on data from [9]. Results show mean ± sem of n = 5–8 animals, the significance of difference between individual groups was determined by post-hoc testing at the P<0.05 levels (*P<0.05). Dtx: Diphtheria treated mice to induce Sertoli cell ablation.

Having previously determined the gross impact of SC ablation on testis development and function at different endpoints [8, 10], we performed a further, more detailed analysis of the LCs. In the adult testis, following ablation of SCs at postnatal day 2 (pnd2-neonatal), the overall tubular architecture was disrupted, and only the tubuli recti and rete testis were maintained due to the retention of SC-like cells in these structures. A large group of ALCs (characterised by large round nucleus and abundant cytoplasm) [11] formed a cluster around the cells of the tubuli recti, near the rete testis (Fig 1A and 1B blue arrows), coinciding with the retention of LC progenitors in the vicinity of the rete testis, as previously described [8]. This observation was confirmed by immunohistochemical staining, demonstrating that these cells expressed CYP11A1 and CYP17A1, both specific markers of LCs in the testis (Fig 1C and 1D red arrows). However, the staining also revealed the presence of another group of LCs further away from the rete testis (Fig 1C and 1D blue arrows). This group of LCs, dispersed in small clusters of 3 or 4 cells exhibited more punctate nuclei and reduced cytoplasmic volume (Fig 1E black arrowhead), similar to the morphology and size of FLCs seen in control neonatal (pnd2) mice (Fig 1F black arrowhead, compare to ALCs in Fig 1A). We suggest that these FLC-like cells are retained, consistent with recent literature showing FLCs are retained through to adulthood [12–15] and further supports the hypothesis that FLCs are independent of the SCs [16]. The cells would however, require further characterisation.

### LC-specific transcript levels are increased following SC ablation in neonatal life

We have previously shown that, despite the impact on ALC number following neonatal ablation of SCs, the endocrine function and regulation of the testis (testosterone and luteinizing hormone (LH) levels) remain largely unaltered in the adult mice [8, 9]. We speculated, therefore, whether functional compensation was occurring in the remaining LC population. In order to test this we quantified the expression of key steroidogenic-related markers by qPCR. Results show that in adult mice the LC-specific transcripts (corrected for LC number), *Lhcgr*, *Cyp11a1* (coding for the rate-limiting enzyme in steroid synthesis), *Hsd3b6*, and *Hsd3b1*, *Star*, *Cyp17a*, *Hsd17b3* (coding for the enzyme required for the final step of testosterone biosynthesis), and *Sult1e1* were significantly increased (Fig 1G) after neonatal ablation of the SCs. This increase in LC-specific transcript levels in adult mice, following neonatal SC ablation, suggests that LC activity is stimulated in these animals. Since transcripts common to both FLC and ALCs as well as adult-specific transcripts (*Hsd3b6*, *Hsd17b3*, *Sult1e1*) are increased it can be inferred that the ALCs are showing increased activity. It is not possible to say whether the FLCs are also affected although previous studies have shown little change in FLC activity in the short-term following SC ablation [9]. Levels of LH are normal in adult animals following neonatal SC ablation suggesting that the LC hyperactivity is due either to altered development of the cells around puberty or to increased activity in the mature cells because of SC loss. A number of factors have been postulated to mediate SC-LC communication [17–23] but definitive studies remain to be carried out.

### Following ablation of SCs in adulthood, the remaining ALCs express functional markers

**Fig 2:**
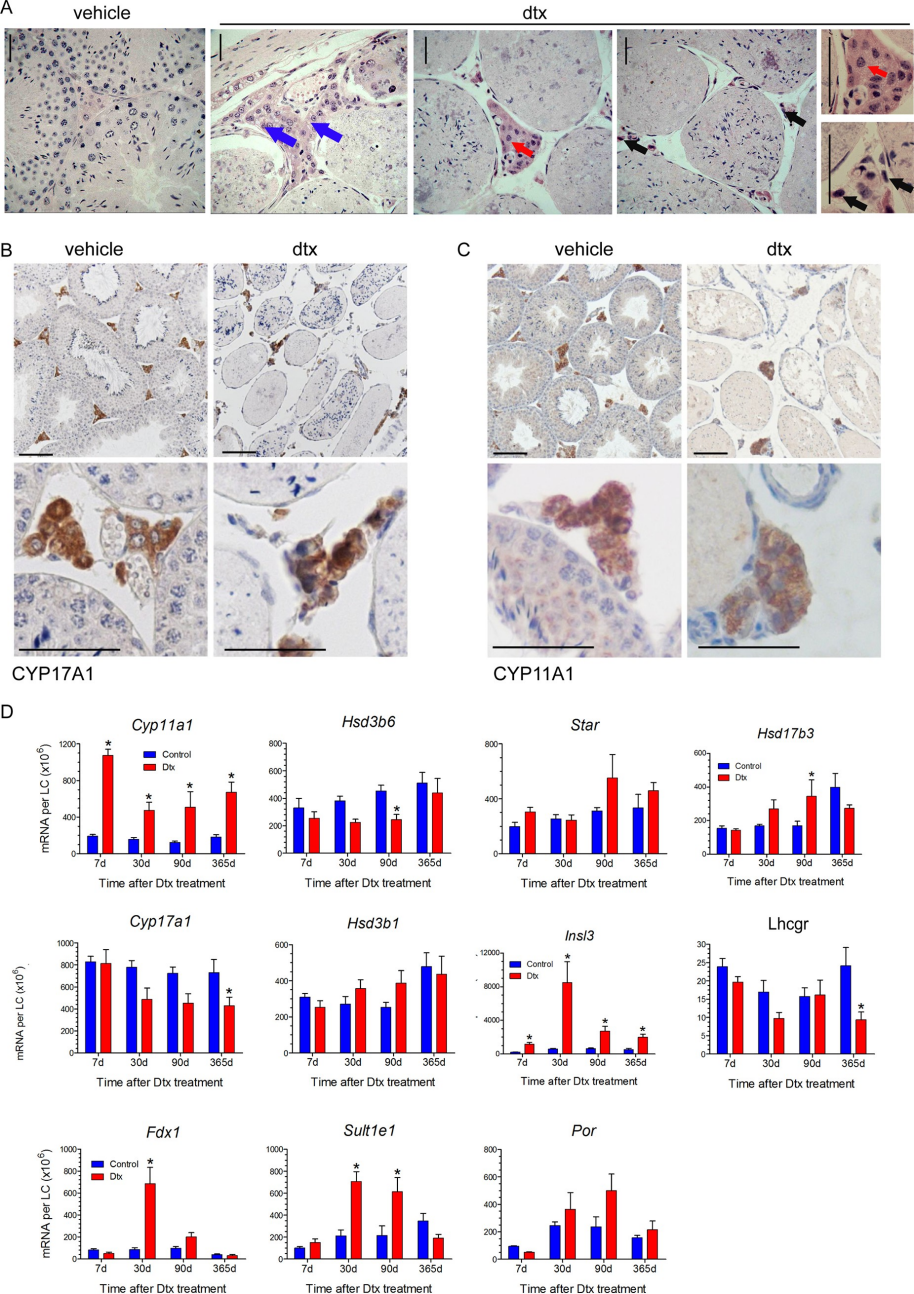
Characterisation of the Leydig cell following Sertoli cell ablation in the adulthood. (A) Adult testis (80 days) 30 days after Sertoli cell ablation at day 50. Large groups of Leydig cells remained present in the subcapsular region of the testis (blue arrows). Some groups of Leydig cell were present in the interstitium (red arrows) but many showed signs of vacuolisation in the cytoplasm (red arrows) but remained generally sparse in most interstitial tissue and those that were present often showed reduced cytoplasmic volume or altered nuclear shape (A black arrows). Immunolocalisation of CYP17A1 (B) and CYP11A1 (C) following Sertoli cell ablation in the adult showed staining within the interstitium although the number of cells stained was much reduced compared to vehicle animals. The black bars represent 50 μm. (D) Sertoli cells were ablated in adult animals at 50 days and transcript levels measured up to 365 days later. Results have been normalised to the number of Leydig cells present in these mice based on data from [8]. Results show mean ± sem of n = 5–8 animals, the significance of difference between individual groups was determined by post-hoc testing at the P<0.05 levels (*P<0.05). Dtx: Diphtheria treated mice to induce Sertoli cell ablation.

Next, we assessed the characteristics of the LCs, which persisted following SC ablation in adulthood (pnd50). Firstly, we re-confirmed the specific localisation of these LCs on the periphery of the testis 30 days after SC ablation, as described previously [8]. Based on morphology, characteristic LCs (large round nucleus, abundant cytoplasm) were identified in the sub-capsular and rete region (Fig 2A-blue arrows) and a mixture of typical LCs as well as vacuolised and pyknotic LCs were visible in the interstitium (Fig 2A-red and black arrows). The remaining “morphologically healthy” LCs in the interstitium are functional as shown with markers such as CYP11A1 and CYP17A1, compared to the pyknotic LCs which do not express these markers (Fig 2B and 2C), which correlates with the endocrine data [8].

### LC-specific transcript levels are altered following SC ablation in adulthood

Following SC ablation in adulthood, circulating testosterone was normal despite only 25% of the LC population being retained [8]. Consequently, we carried out a transcript analysis to assess whether the remaining 25% LCs were also functionally compensating for the reduction in LC population size. Results corrected for LC number show that *Cyp11a1* and *Insl3* transcripts were significantly increased at all-time points following SC ablation while *Hsd17b3*, *Fdx1 and Sult1e1* were significantly increased at one or more times. Only *Hsd3b6*, *Cyp17a1* and *Lhcgr* transcript levels decreased following adult SC ablation (Fig 2D). These results show that LCs undergo an adaptive response in transcription relating to function in the absence of SCs in adulthood. This may be different to the compensation mechanism observed when SC are ablated in neonatal life and may reflect hypersensitivity of the LCs to LH stimulation. Increased LC steroidogenesis was also seen following SC ablation in adult animals, indicative of the functional compensation of the remaining 25% of cell [8, 9]. This may be a consequence of an altered endocrine environment for the cells as SC ablation leads to dysfunction in the testicular vascular network [24]. It is also possible however, that the SC-independent LCs, which remain after SC ablation, are functionally different to the other 75% of cells which undergo apoptosis in the absence of the SCs [8].

### Could LC transcripts expression reflect LC cell number?

**Fig 3:**
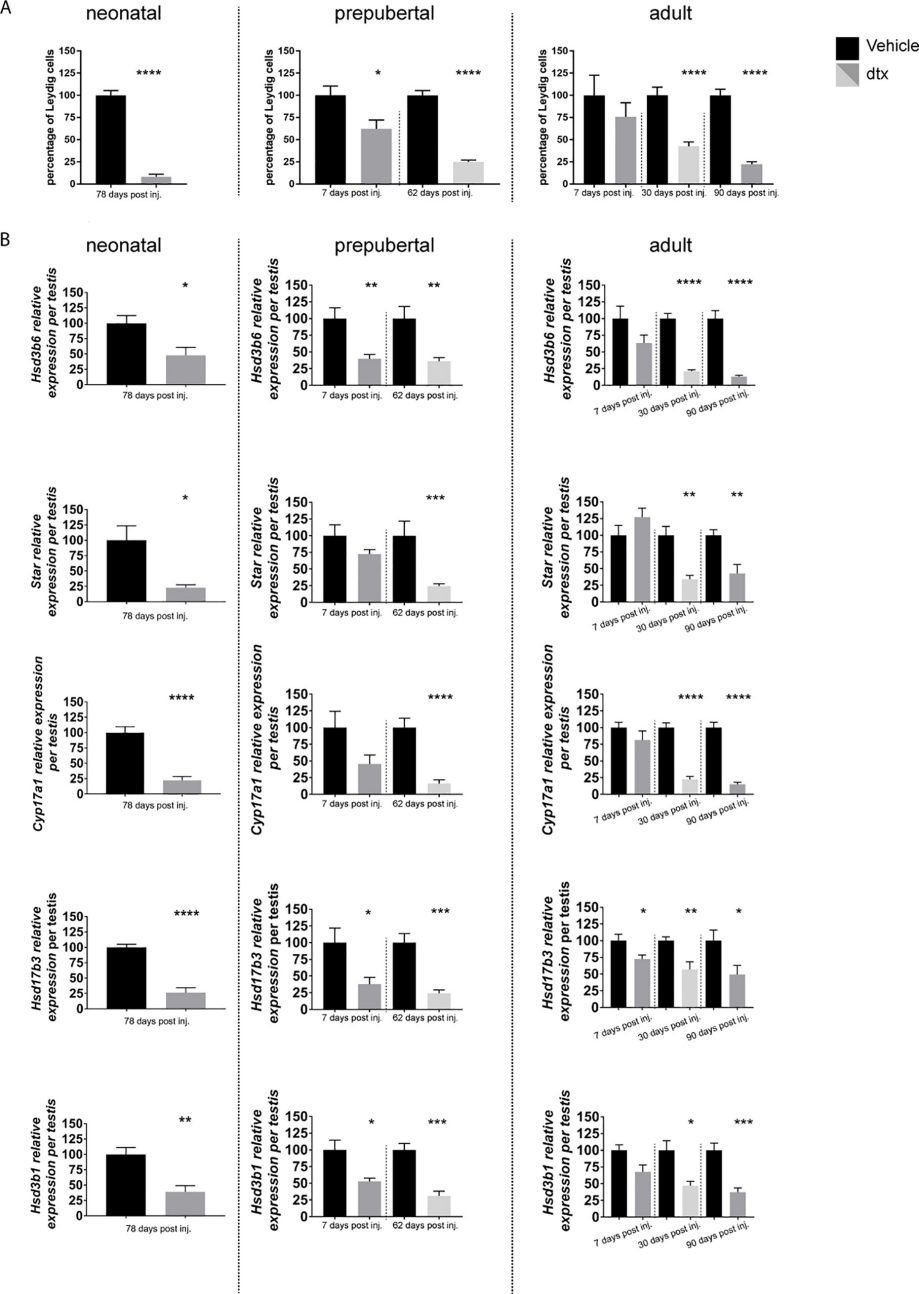
Identification of Leydig cell markers reflecting the cell number. (A) Leydig cells number following Sertoli cell ablation in neonatal, prepubertal and adult life, this data, previoulsy published, is shown again here in percentages to allow direct comparison with transcript levels [8, 9]. (B) Transcript levels measured at the same endpoints as (A) following Sertoli cell ablation in neonatal, prepubertal and adult life. Results have been normalised to an external control and represent transcript levels per testis. Results show mean ± sem of n = 5–8 animals, the significance of difference between individual groups was determined by post-hoc testing at the P<0.05 levels (*P<0.05). Dtx: Diphtheria treated mice to induce Sertoli cell ablation.

Our next interrogation focussed on whether we could identify transcripts which would reflect the number of LCs in the adult testis, as a shorthand way of assessing whether further stereological analysis was required. Thus, we used models where the reduction of the ALC number was associated with either a decrease in their progenitor cell recruitment (neonatal ablation) or a survival defect (prepubertal and adult SC ablation). Moreover, to avoid any misinterpretation, with regards to the development and function of the ALCs, which are closely related to LH and androgen stimulation, [11, 25], we set our study in a context where both LH and testosterone levels are normal but where LC are functionally overactive [8, 9]. To identify candidates, we collated our results on LC number previously obtained following neonatal, prepubertal and adult SC ablation and published [8, 9] (shown again here to allow direct comparison with transcript levels and expressed in percentages-Fig 3A). To determine whether any of the Leydig cell markers could act as a proxy for Leydig cell numbers, transcript levels were measured per testis and expressed relative to the external standard (luciferase mRNA) (Fig 3B). Only *Hsd3b6*, *Star*, *Cyp17a1*, *Hsd17b3*, *Hsd3b1* were significantly reduced following SC ablation and could, therefore, potentially act a marker of LC number (Fig 3B). Of those transcripts, however, only *Hsd3b1* has been shown to be unresponsive to LH/hCG stimulation [26], identifying this gene as a potential candidate biomarker for LC population size.

To further confirm the validity of this candidate, we collated data from previous models (S2 Table) such as the SC specific androgen knock-out model (SCARKO) and ethane dimethane sulphonate (EDS) models where LC number is reduced and testosterone and LH levels are normal [27, 28]. Within the limits of gene expression examined in each study, we confirmed that transcript *Hsd3b1* is a good indicator of the ALC number in all of these models.

ALC development, maturation and function can be regulated by multiple factors [25] but, for this study, we selected models where the change in LC number came from either altered progenitor recruitment or a change in LC survival and where the hormonal background was not different to control animals [29]. Initially, 5 candidate transcripts were identified, all independent of germ cell regulation [30]. Previous data from *hpg* mice have indicated however, that most transcripts respond to LH/hCG stimulation with the exception of *Hsd3b1* [26, 31, 32].

## Limitations

This study identifies *Hsd3b1* transcript levels as a potential indicator of LC number with utility for informing decision-making on whether to engage in time-consuming stereological cell counting analysis. As HSD3B1 is integral to the principal steroidogenic function of the Leydig cells it is possible that it may be under endocrine or paracrine control not considered here. Measurement of transcript levels reported here was based on using an external standard for the qPCR. This may not be possible in all circumstances and use of housekeeping genes to normalise qPCR results may make interpretation of the data more complex.

## Materials and methods

### Animals and treatments

All animal studies passed local ethical review and were conducted with licenced permission under the UK Animal Scientific Procedures Act (1986), Home Office licence number PPL 60/4200. Amh-Cre; iDTR mice were generated, genotyped and treated as previously described [11,12].

### RNA extraction, reverse transcription and real-time PCR

Total RNA extraction, reverse transcription, primer design and real-time PCR were carried out as previously described [11,12][15,16]. RNA was extracted using TRIzol (Life Technologies, Paisley, UK). Luciferase mRNA (5ng per testis, Promega UK, Southampton, UK) was added as an external standard to each sample at the start of the RNA extraction procedure. This controls for the efficiency of RNA extraction, RNA degradation, and the reverse transcription step and allows specific transcript levels to be expressed per testis. Isolated RNA was reverse transcribed using random hexamers and Moloney murine leukemia virus reverse transcriptase (Superscript III; Life Technologies). For real-time PCR, SYBR mastermix (Brilliant II, Agilent, Amsterdam, The Netherlands) was mixed with primer (100 nM) and template in a total volume of 10μl and amplified with an Agilent MX3000 cycler. All primers were designed by PrimerExpress 2.0 (Applied Biosystems, Warrington, UK). Transcript levels were normalised relative to the luciferase external standard, generating a value of transcript expression per testis. The primers used are described in S1 Table.

### Histology and immunohistochemistry

For histology testes were fixed for 6 hours in Bouin’s solution and then embedded in Technovit 7100 resin. Sections (2.5μm) were cut and stained with H&E. Immunohistochemistry was carried out as described [11,12]; testes were embedded in paraffin and sections (5μm) prepared and stained using anti-CYP11A1 (rabbit polyclonal, donated by Dr AH Payne, Stanford University) diluted 1/500 or anti-CYP17A1 (sc-46081 Santa Cruz, lot number F2014, Heidelberg, Germany) diluted 1/400 and using the appropriate secondary antibodies conjugated to biotin, diluted at 1/500 in the blocking serum. This step was followed by incubation with horseradish peroxidase labelled avidin-biotin complex (VectorLabs, Peterborough, UK). The revelation was undertaken with diaminobenzidine DAB (Immpact DAB;VectorLabs, Peterborough, UK). Slides were counterstained with haematoxylin, dehydrated and mounted with Pertex mounting medium (Cell Path, Hemel Hempstead, UK). Cell numbers were measured by the optical dissector as previously shown [8, 9]. The cells were recognised by their position, round nucleus and relatively abundant cytoplasm. The total testis volume was estimated using the Cavalieri principle.

### Statistics

Data sets were analysed using General Linear Modelling. Where this showed a significant overall difference, the significance of difference between individual groups was determined by post-hoc testing at the P<0.05 levels. Data was log transformed where appropriate to avoid heterogeneity of variance. Data was analysed using Minitab 15 (Minitab Ltd, Coventry, UK) or Graph Prism version 8 (GraphPad Software Inc., San Diego, CA, USA).

## Supporting information

S1 Table Primer list. (XLSX)

S2 Table List of models exhibiting Leydig cell number decrease. (PDF)
