# Supplementary material for: Relationship of transcriptional markers to Leydig cell number in the mouse testis
Source: PLoS One. 2019 Jul 10;14(7):e0219524. doi: 10.1371/journal.pone.0219524 (PMC6619764; doi:10.1371/journal.pone.0219524)
Supplement: S2 Table — (PDF) [file pone.0219524.s002.pdf]

S2 table

Model	refs	LC nb	[T]	[LH]	*Star*	*Cyp11a1*	*Hsd3b1*	*Cyp17a1*	*Hsd3b6*	*Hsd17b3*	*Insl3*	*EST*
EDS	Zhang YF, Yuan KM, Liang Y, Chu YH, Lian QQ, Ge YF, et al. Alterations of gene profiles in Leydig-cell-regenerating adult rat testis after ethane dimethane sulfonate-treatment. Asian J Androl. 2015;17(2):253-60.	Reduced 35%	Normal	?	Normal per rps16	Normal per rps16	Normal per rps16	Normal per rps16	Normal per rps16	Normal per rps16	Normal per rps16	nd
	O'Shaughnessy PJ, Hu L, Baker PJ. Effect of germ cell depletion on levels of specific mRNA transcripts in mouse Sertoli cells and Leydig cells. Reproduction. 2008;135(6):839-50.	?	Reduced	?	nd	Normal per testis	Normal per testis	Normal per testis	?	nd		
SCARKO	De Gendt K, Atanassova N, Tan KA, de Franca LR, Parreira GG, McKinnell C, et al. Development and function of the adult generation of Leydig cells in mice with Sertoli cell-selective or total ablation of the androgen receptor. Endocrinology. 2005;146(9):4117-26.	Reduced ~40%	Normal	Normal	nd	Increased mRNA/LC nb	Increased mRNA/LC nb	Increased mRNA/LC nb	nd	nd	Normal mRNA/LC nb	Increased mRNA/LC nb
ARKO	De Gendt K, Atanassova N, Tan KA, de Franca LR, Parreira GG, McKinnell C, et al. Development and function of the adult generation of Leydig cells in mice with Sertoli cell-selective or total ablation of the androgen receptor. Endocrinology. 2005;146(9):4117-26.	Reduced ~80%	Normal	Increased	nd	Increased mRNA/LC nb	Increased mRNA/LC nb	Decreased mRNA/LC nb	nd	nd	Decreased mRNA/LC nb	Decreased mRNA/LC nb
	O'Shaughnessy PJ, Monteiro A, Abel M. Testicular development in mice lacking receptors for follicle stimulating hormone and androgen. PLoS One. 2012;7(4):e35136.	Reduced	nd	nd	nd	nd	nd	nd	nd	nd	nd	nd
	O'Shaughnessy PJ, Johnston H, Willerton L, Baker PJ. Failure of normal adult Leydig cell development in androgen-receptor-deficient mice. J Cell Sci. 2002;115(Pt 17):3491-6	Reduced	nd	nd	Normal per testis	Reduced per testis	Normal per testis	Reduced per testis	Reduced per testis	Reduced per testis		Reduced per testis
FSHRKO	Baker PJ, Pakarinen P, Huhtaniemi IT, Abel MH, Charlton HM, Kumar TR, et al. Failure of normal Leydig cell development in follicle-stimulating hormone (FSH) receptor-deficient mice, but not FSHbeta-deficient mice: role for constitutive FSH receptor activity. Endocrinology. 2003;144(1):138-45.	Reduced	Normal Reduced itt	Increased	Reduced per testis	Reduced per testis	nd	Normal per testis	Reduced per testis	Normal per testis	nd	nd
	O'Shaughnessy PJ, Monteiro A, Abel M. Testicular development in mice lacking receptors for follicle stimulating hormone and androgen. PLoS One. 2012;7(4):e35136.	Reduced	nd	nd	nd	nd	nd	nd	nd	nd	nd	nd
FSHRKO+ARKO	O'Shaughnessy PJ, Monteiro A, Abel M. Testicular development in mice lacking receptors for follicle stimulating hormone and androgen. PLoS One. 2012;7(4):e35136.	Reduced	nd	nd	nd	nd	nd	nd	nd	nd	nd	nd
MIS-tg	Racine C, Rey R, Forest MG, Louis F, Ferre A, Huhtaniemi I, et al. Receptors for anti-mullerian hormone on Leydig cells are responsible for its effects on steroidogenesis and cell differentiation. Proc Natl Acad Sci U S A. 1998;95(2):594-9.	Reduced	Reduced	Increased	nd	nd	nd	Reduced	nd	nd	nd	nd
MISKO		27% of males show hyperplasia	Normal	Normal	nd	nd	nd	increased	nd	nd	nd	nd

nd: non determined

EDS: Leydig cell ablation with ethane dimethane sulfonate-treatment

SCARKO: Sertoli cell androgen receptor knock-out

ARKO: Androgen receptor knock-out

FSHRKO: Follicle stimulating hormone receptor knock out

MIS tg: overexpression of anti-Müllerian hormone

MISKO: knock-out of anti-Müllerian hormone
